# Groups without Cultured Representatives Dominate Eukaryotic Picophytoplankton in the Oligotrophic South East Pacific Ocean

**DOI:** 10.1371/journal.pone.0007657

**Published:** 2009-10-29

**Authors:** Xiao Li Shi, Dominique Marie, Ludwig Jardillier, David J. Scanlan, Daniel Vaulot

**Affiliations:** 1 UPMC (Paris-06) and CNRS, UMR 7144, Station Biologique de Roscoff, Roscoff, France; 2 Department of Biological Sciences, University of Warwick, Coventry, United Kingdom; 3 Nanjing Institute of Geography and Limnology, Chinese Academy of Sciences, Nanjing, People's Republic of China; University of Poitiers, France

## Abstract

**Background:**

Photosynthetic picoeukaryotes (PPE) with a cell size less than 3 µm play a critical role in oceanic primary production. In recent years, the composition of marine picoeukaryote communities has been intensively investigated by molecular approaches, but their photosynthetic fraction remains poorly characterized. This is largely because the classical approach that relies on constructing 18S rRNA gene clone libraries from filtered seawater samples using universal eukaryotic primers is heavily biased toward heterotrophs, especially alveolates and stramenopiles, despite the fact that autotrophic cells in general outnumber heterotrophic ones in the euphotic zone.

**Methodology/Principal Findings:**

In order to better assess the composition of the eukaryotic picophytoplankton in the South East Pacific Ocean, encompassing the most oligotrophic oceanic regions on earth, we used a novel approach based on flow cytometry sorting followed by construction of 18S rRNA gene clone libraries. This strategy dramatically increased the recovery of sequences from putative autotrophic groups. The composition of the PPE community appeared highly variable both vertically down the water column and horizontally across the South East Pacific Ocean. In the central gyre, uncultivated lineages dominated: a recently discovered clade of Prasinophyceae (IX), clades of marine Chrysophyceae and Haptophyta, the latter division containing a potentially new class besides Prymnesiophyceae and Pavlophyceae. In contrast, on the edge of the gyre and in the coastal Chilean upwelling, groups with cultivated representatives (Prasinophyceae clade VII and Mamiellales) dominated.

**Conclusions/Significance:**

Our data demonstrate that a very large fraction of the eukaryotic picophytoplankton still escapes cultivation. The use of flow cytometry sorting should prove very useful to better characterize specific plankton populations by molecular approaches such as gene cloning or metagenomics, and also to obtain into culture strains representative of these novel groups.

## Introduction

Photosynthetic picoeukaryotes (PPE), with a cell size less than 2–3 µm, play a critical role in oceanic primary production [1]. Molecular approaches have led to significant progress in our assessment of the composition and distribution of marine picoeukaryote communities. In particular, the analysis of 18S rRNA gene diversity from picoplankton samples led to the discovery of numerous new groups within the heterotrophs [2]–[4]. More specifically, many marine picoplankton sequences can be attributed to alveolates (Syndiniales group I and II in particular [5]), many of which are probably parasites of larger phytoplankton species [6], or to heterotrophic stramenopiles [7], which in contrast to alveolates are probably mostly predators [8]. However, the fraction of 18S rRNA gene sequences from photosynthetic picoplankton relative to heterotrophic ones remains low [9] and little diversified, despite the larger relative abundance of autotrophic cells observed in the euphotic zone in eutrophic and mesotrophic regions [10]. Although very few picophytoplanktonic eukaryotic species have been described to date [9], 18S rRNA gene clone libraries constructed from filtered samples have not suggested the existence of uncultured groups with the notable exception of picobiliphytes which seems to have affinities with cryptophytes [11]. In contrast, most novel photosynthetic groups have been discovered through cultures, such as the Bolidophyceae [12] or the Pinguiophyceae [13]. These data raised the possibility that photosynthetic picoeukaryotes were indeed very little diversified, as is the case for marine picoplanktonic cyanobacteria dominated by only two closely related genera *Prochlorococcus* and *Synechococcus* [14], [15].

However two major strategies have been developed in recent years to target more specifically PPE diversity, bringing in new data. Firstly, analysis of the plastid 16S rRNA gene has suggested that Chrysophyceae, a class whose autotrophic members were thought to be restricted to freshwater, and Prymnesiophyceae, a class known to be important in oceanic waters through its diagnostic pigment 19'hexanoyloxyfucoxanthin but for which very few sequences have been recovered from picoplankton [16], could be important PPE contributors and highly diversified [17], [18]. Secondly, the use of 18S rRNA gene primer sets biased towards Chlorophyta uncovered novel prasinophyte lineages (clades VIII and IX) in the Mediterranean Sea and detected a much wider diversity at lower taxonomic levels (genus) than could be obtained with universal primers [19]. However, these two approaches suffer from limitations. For the first one, the number of plastid 16S rRNA gene sequences available for known photosynthetic species is much smaller than for the 18S rRNA gene, making sequence assignment much more uncertain. For the second approach, biased 18S rRNA gene primers only target a fraction of the photosynthetic taxa, e.g. only the green algal lineage (Chlorophyta), and one cannot expect to obtain a complete image of environmental PPE diversity.

Flow cytometry has been used for quite a long time to estimate PPE abundance in the field, allowing for example derivation of macro-ecological patterns [20]. However, its sorting capacity has been surprisingly little used to collect information about PPE (but see [1], [21]). This could be explained in part by the complexity and slow sorting rate of previously available instruments. Recently, the advent of compact high-speed sorters that can be taken on board ship has offered novel opportunities. We developed a protocol to concentrate cells by tangential flow filtration, sort PPE by flow cytometry, and construct 18S rRNA gene clone libraries using universal primers. This protocol was tested on samples from the English Channel, demonstrating that the resulting clone libraries were highly enriched in photosynthetic organisms [22]. In the present paper, we applied the same approach using on-board flow cytometry during the BIOSOPE cruise throughout the South East Pacific. This oceanic region, which has been very little sampled, is of special interest because it offers extreme trophic gradients [23] from nutrient-rich coastal upwelling waters off Chile to the crystal-clear waters off Easter Island [24]. The western side of the gyre is characterized by high nutrient low chlorophyll (HNLC) waters close to the equator. Our data confirm that PPE are highly diversified and demonstrate the existence of many uncultured groups, especially in the oligotrophic central gyre. They complement data obtained during the same cruise on PPE using 16S rRNA plastid genes amplified from <3 µm filtered samples [25].

## Results and Discussion

### 18S rRNA Gene Clone Libraries from Sorted PPE

**Figure 1:**
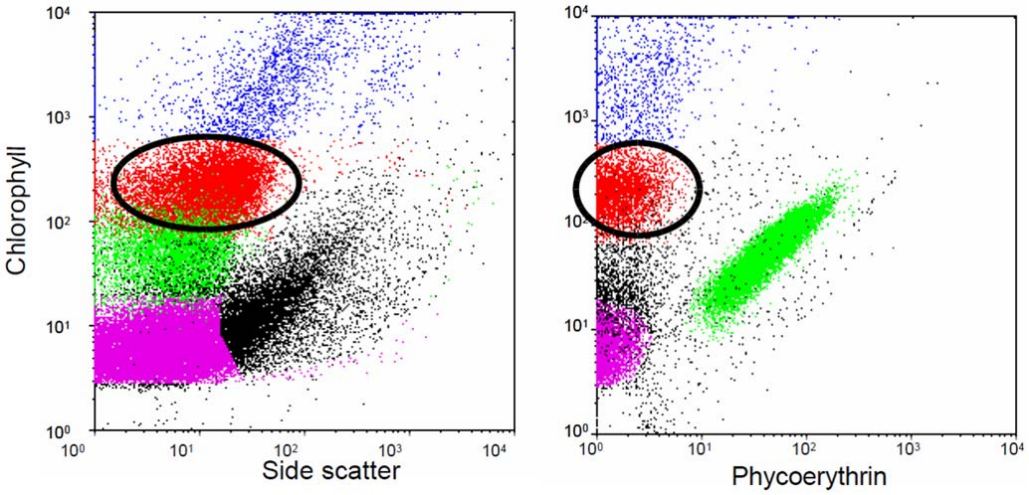
Flow cytometry analysis (side scatter vs. red chlorophyll fluorescence and orange phycoerythrin vs. red chlorophyll fluorescence) of photosynthetic plankton from BIOSOPE Station STB17 at 70 m (sorted sample T120). The red circled population corresponds to photosynthetic pico-eukaryotes that have been targeted for sorting. The magenta, green and blue populations correspond to the cyanobacteria *Prochlorococcus* and *Synechococcus* and to the nano-eukaryotes respectively.

**Figure 2:**
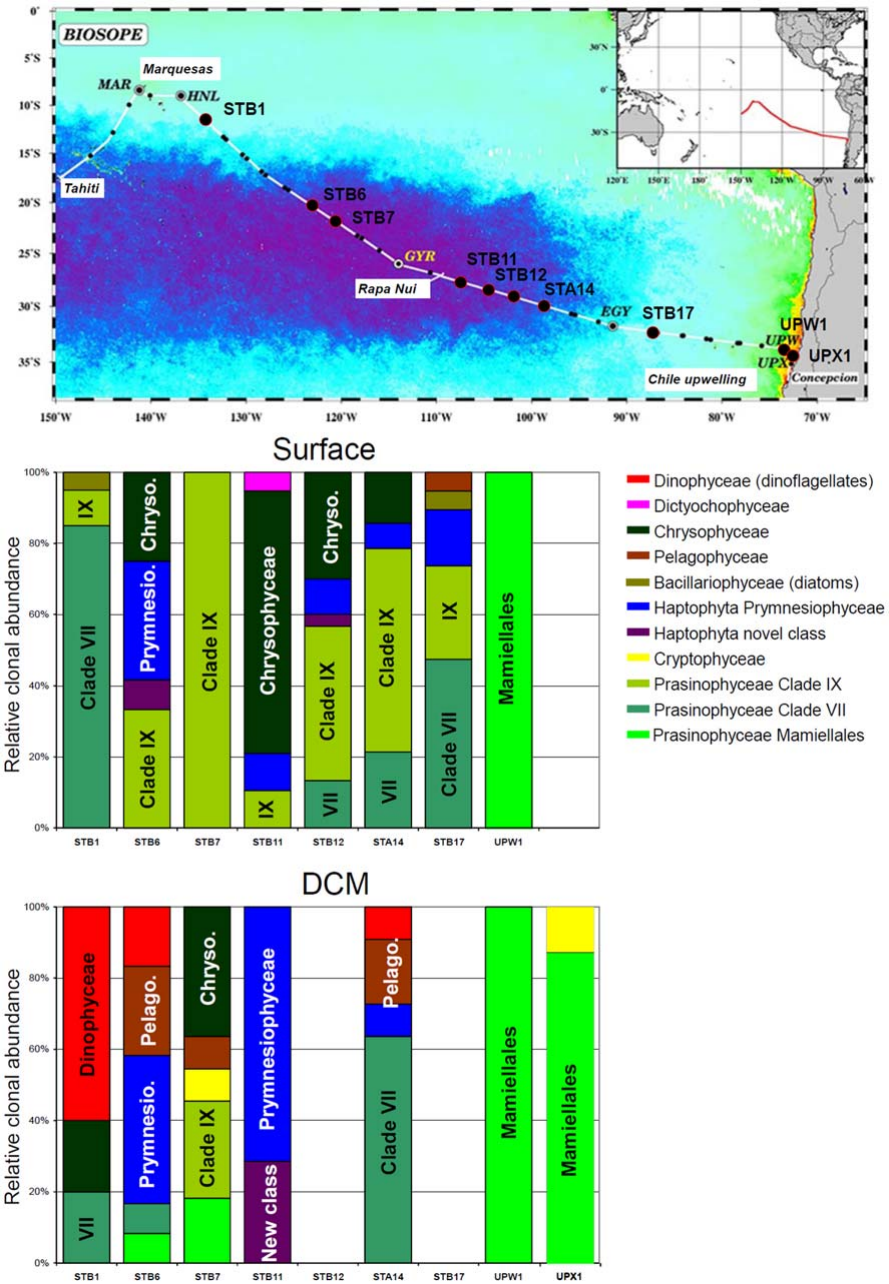
BIOSOPE cruise track map superimposed on a SeaWiFS ocean colour composite, the dark purple indicating extremely low values (0.018 mg m^−3^) of total chlorophyll *a*. Bar charts represent the taxonomic composition of the PPE community based on 18S rRNA gene sequences obtained from PPE sorted samples at the different stations in surface waters and at the deep chlorophyll maximum (DCM).

We characterized PPE populations by flow cytometry (Fig. 1) using samples collected in surface waters and near the deep chlorophyll maximum (DCM) along a transect through the South East Pacific Ocean (Fig. 2, Table 1) during the 2004 BIOSOPE cruise [23]. PPE abundance ranged from 600 to 37,000 cell mL^−1^ with maximum numbers in the Chilean upwelling and lowest values in the center of the gyre [26]. After concentration by tangential flow filtration [22], from 80,000 to 500,000 PPE cells were sorted by flow cytometry (Table 1) and 18S rRNA gene clone libraries were constructed using universal primers.

**Table 1:** BIOSOPE sample locations, PPE abundance (from [26]), and clone library information. Oligo = oligotrophic, Meso = mesotrophic and Eutro = eutrophic.

Station	Longitude (°)	Latitude (°)	Trophic status	Sample Code	Depth (m)	PPE concentration (cell mL^−1^)	Number of PPE cells sorted	Nested PCR	Primer used for partial sequence	Number of partial sequences	Number of full sequences
STB1	−134.10	−11.74	Meso	T17	90	4 049	91 407		Euk528f	13	0
				T19	25	7 017	103 074	Yes	Euk528f	20	6
STB6	−122.89	−20.45	Oligo	T33	180	2 453	225 500		Euk528f	29	5
				T35	55	615	151 900	Yes	Euk528f	26	0
STB7	−120.38	−22.05	Oligo	T39	175	919	106 000		Euk528f	37	9
				T41	40	791	125 000		Euk528f	22	11
STB11	−107.29	−27.77	Oligo	T58	200	1 519	168 000		Euk528f	21	3
				T60	0	1 305	171 440	Yes	Euk528f	19	4
STB12	−104.31	−28.54	Oligo	T65	40	861	123 000	Yes	EukA	33	11
STA14	−98.39	−30.04	Oligo	T84	5	1 007	118 000		Euk528f	22	4
				T88	150	3 974	236 500		Euk528f	19	2
STB17	−86.78	−32.40	Meso	T123	20	6 802	124 000		Euk528f	24	4
UPW1	−73.37	−34.00	Eutro	T142	5	1 732	104 000	Yes	Euk528f	17	1
				T148	35	4 820	81 000		Euk528f	17	1
UPX1	−72.42	−34.51	Eutro	T159	40	37 539	507 000	Yes	EukA	27	0

Overall, we obtained 413 partial 18S rRNA gene sequences. Among these, we detected at least 12 chimeras, often between closely related sequences (e.g. between two Mamiellales, Table S1) as observed in sorted samples from the English Channel [22]. Fifty one sequences corresponded to fungi and were related to common laboratory contaminants. This contamination probably occurred during DNA extraction or PCR amplification back in the laboratory and came to the surface because of the very low DNA quantities in the sorted populations. Indeed 34 out of 51 fungal sequences were closely related to *Sporobolomyces roseus* which was also found contaminating English Channel sorted samples [22]. We also obtained four metazoan sequences related to copepods that could originate from eggs or debris that may have been sorted in the same drop as a PPE. Of the remaining 346 sequences, 223 (64.5%) belonged to putative photosynthetic groups (Table 2, Table S2) and the rest to heterotrophic protists, mostly alveolates (Syndiniales groups I, II, and III, [5]) and stramenopiles. The high proportion of photosynthetic sequences recovered compared to what is usually obtained for filtered samples (on average 30%, [9]) proves that flow cytometry sorting was efficient to separate autotrophs from heterotrophs confirming a parallel study [22]. Sequences from heterotrophic protists that are known to be parasitic, such as the Syndiniales, could originate from parasites carried by the PPE cells themselves. In contrast sequences from heterotrophs that are likely to be phagotrophic (e.g. stramenopiles or *Telonema*) could come from predating cells that had engulfed PPE cells immediately prior to sorting and therefore presented similar fluorescence signals. Another possibility is that an undetected non-photosynthetic cell may be sorted in the same drop as a photosynthetic one. Chimeric sequences as well as those from fungi, metazoans and heterotrophic protists are not further considered.

**Table 2:** Number of partial sequences obtained for each sample and each taxonomic group (Fungi, Metazoa and chimeras excluded).

Sample				T17	T19	T33	T35	T39	T41	T58	T60	T65	T84	T88	T123	T142	T148	T159
Station				STB1	STB1	STB6	STB6	STB7	STB7	STB11	STB11	STB12	STA14	STB14	STB17	UPW1	UPW1	UPX1
Depth (m)				90	25	180	55	175	40	200	0	40	5	150	20	5	35	40
Taxonomy	Photosynthetic	Total	%															
Chlorophyta/Prasinophyceae/Mamiellales	Yes	45	13.0%			1		2								17	4	21
Chlorophyta/Prasinophyceae/Clade VII	Yes	42	12.1%	1	17	1						4	3	7	9			
Chlorophyta/Prasinophyceae/Clade IX	Yes	55	15.9%		2		4	3	18		2	13	8		5			
Cryptophyta/Cryptophyceae	Yes	4	1.2%					1										3
Haptophyta/Novel class	Yes	4	1.2%				1			2		1						
Haptophyta/Prymnesiophyceae	Yes	24	6.9%			5	4			5	2	3	1	1	3			
Stramenopiles/Bacillariophyceae	Yes	2	0.6%		1										1			
Stramenopiles/Pelagophyceae	Yes	7	2.0%			3		1						2	1			
Stramenopiles/Chrysophyceae	Yes	33	9.5%	1			3	4			14	9	2					
Stramenopiles/Dictyochophyceae	Yes	1	0.3%								1							
Stramenopiles/Bicosoecida		3	0.9%					2							1			
Stramenopiles/MAST IV		2	0.6%	1									1					
Stramenopiles/Oomycetes		1	0.3%						1									
Stramenopiles																		
Alveolata/Dinophyceae	Yes	6	1.7%	3		2								1				
Alveolata/group I		12	3.5%	1		1		4					1	1			4	
Alveolata/group II		88	25.4%	6		10	14	18	3	10		3	6	3	3		9	3
Alveolata/group III		10	2.9%			1		1		4				3	1			
Radiolaria		6	1.7%			4		1						1				
Telonemia		1	0.3%			1												
Chlorophyta		142	41.0%	1	19	2	4	5	18		2	17	11	7	14	17	4	21
Cryptophyta		4	1.2%					1										3
Haptophyta		28	8.1%			5	5			7	2	4	1	1	3			
Stramenopiles		49	14.2%	2	1	3	3	7	1		15	9	3	2	3			
Alveolata		116	33.5%	10		14	14	23	3	14		3	7	8	4		13	3
Photosynthetic (potentially) sequences		223	64.5%	5	20	12	12	11	18	7	19	30	14	11	19	17	4	24
All sequences		**346**		**13**	**20**	**29**	**26**	**37**	**22**	**21**	**19**	**33**	**22**	**19**	**24**	**17**	**17**	**27**

### Diversity of Photosynthetic PPE

Sequences of the 18S rRNA gene from photosynthetic groups were mainly affiliated to Prasinophyceae, Chrysophyceae, and Haptophyta, which matches the data obtained during the same cruise on the plastid 16S rRNA gene of PPE [25]. A limited number of sequences belonged to other photosynthetic stramenopiles classes, to Cryptophyta and to Dinophyceae (Table 2). Sequences were grouped into 79 operational taxonomic units (OTUs, Table S2), using a 98% sequence identity cut-off level consistent with our previous work [27] and corresponding to the average similarity threshold at the species level for eukaryotic microbes [28]. Many OTUs were only distantly related to known groups, highlighting the high diversity recovered by this approach. Full length sequences representative of OTUs without closely related cultivated species were obtained (Table S3) in order to perform more detailed phylogenetic analyses of these novel groups.

**Figure 3:**
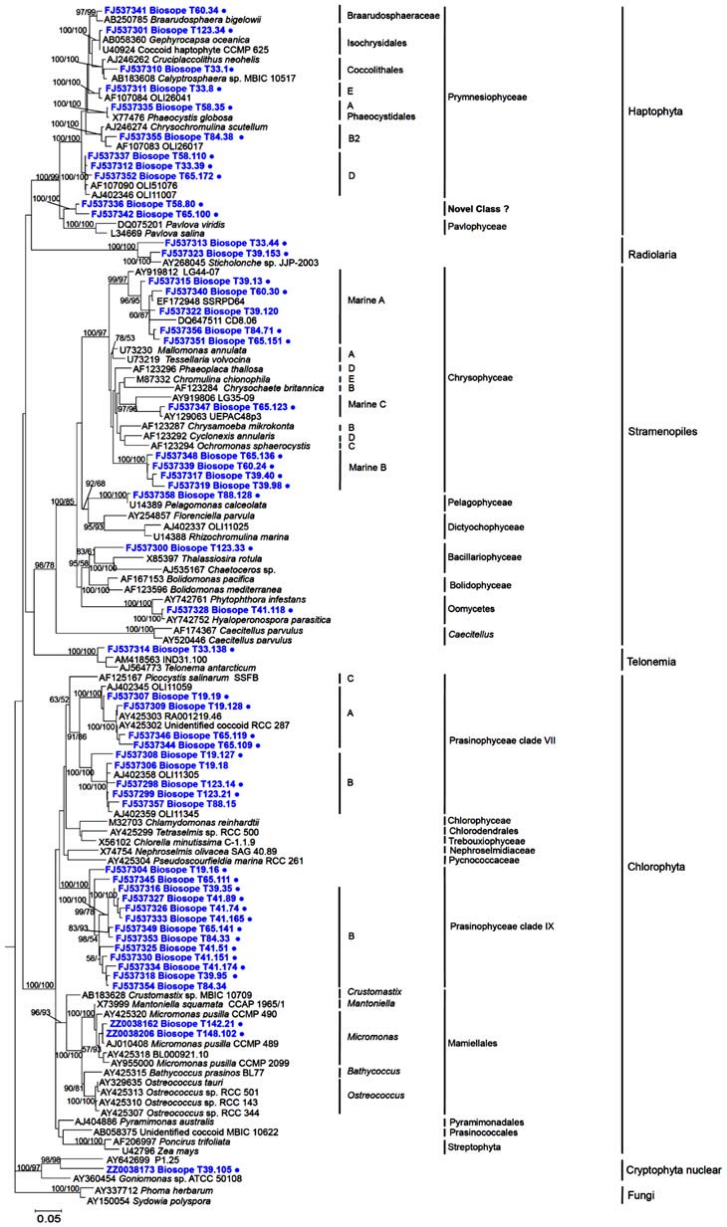
Phylogenetic tree of 18S rRNA gene sequences retrieved from PPEs sorted by flow cytometry in the South East Pacific (in bold blue) inferred by maximum likelihood (ML) analysis. Sequences representative of OTUs are labelled with a dot. Clade nomenclatures follow references [19], [29] for Prasinophyceae, [51] for Chrysophyceae and [32] for Haptophyta. The tree is inferred from 1,622 positions of an alignment of 124 full-length sequences with two outgroup sequences (fungi). The phylogenetic tree was based on a TrN+I+G model of DNA substitution with a gamma distribution shape parameter of 0.5833 and substitution rates of R(b)[A–G] = 2.5256, R(e)[C–T] = 4.3865 and 1.0 for all other substitution rates. The total number of rearrangements tried was 70,412. Bootstrap values over 50% are indicated on the internal branches obtained from both NJ and MP methods.

**Figure 4:**
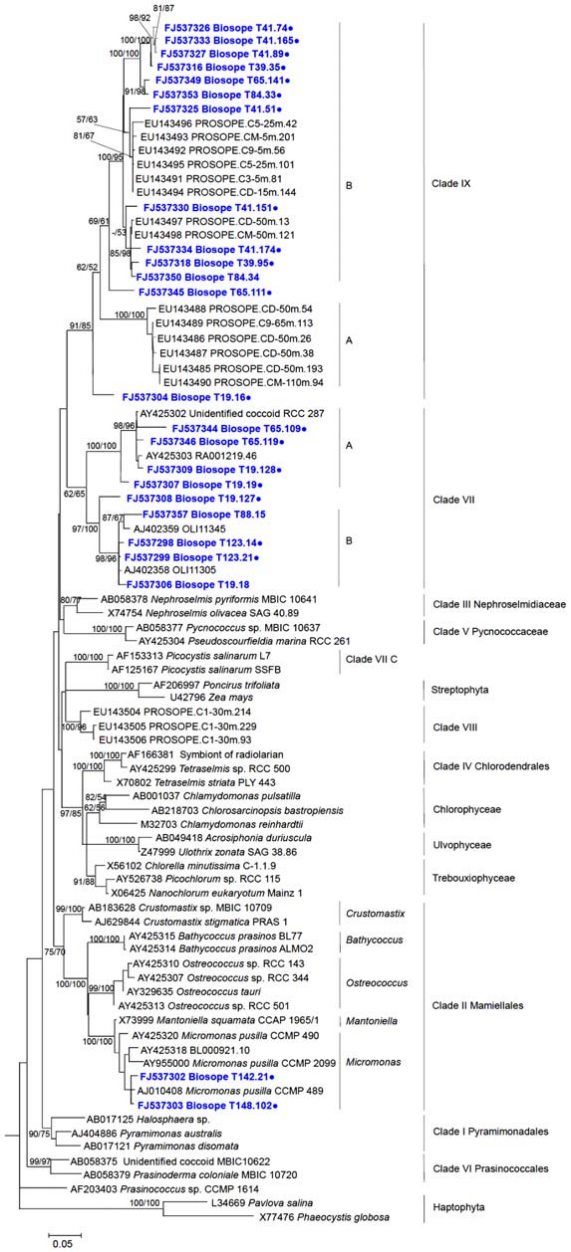
Phylogenetic tree of 18S rRNA gene sequences for Prasinophyceae retrieved from PPEs sorted by flow cytometry in the South East Pacific (in bold blue) inferred by ML analysis. Sequences representative of OTUs are labelled with a dot. 888 positions of an alignment of 85 partial sequences were used. The phylogenetic tree was based on a TrN+I+G model of DNA substitution with a gamma distribution shape parameter of 0.5547 and substitution rates of R(b)[A–G] = 2.3180, R(e)[C–T] = 4.9754 and 1.0 for all other substitution rates. Total number of rearrangements tried was 75,853. Bootstrap values over 50% are indicated on the internal branches obtained from both NJ and MP methods.

Among the Prasinophyceae, the most interesting group comprised 12 OTUs (for which we obtained 17 full length sequences) originating almost exclusively from oligotrophic stations (STB6 to STB14, Fig. 2) that appeared to form an independent cluster (Fig. 3). BLAST analyses revealed that some of these sequences were closely related to sequences of Prasinophyceae clade IX (see Table S3) recently retrieved from picoplankton at pelagic Mediterranean Sea stations using the Chloroplastida biased primer CHLO02 [19]. However, use of this latter primer only allowed retrieval of partial sequences (roughly 800 bp) in contrast to our approach which provides full length sequences. Phylogenetic analysis of the region of overlap between the Pacific and Mediterranean sequences confirmed that Pacific sequences indeed belonged to Prasinophyceae clade IX. Most of them fell more precisely into sub-clade IX-B with high bootstrap support and two sequences (T65.111 and T19.16) could not be assigned to any sub-clade (Fig. 3, Fig. 4).

Another large group of 7 OTUs (10 full sequences), originating from both oligotrophic and mesotrophic regions, was affiliated to Prasinophyceae Clade VII, a group previously divided into three sub-clades [29]. While sub-clade C corresponds to *Picocystis salinarum*, a species originating from a hyper-saline lake having probably a very restricted ecological range, sequences from sub-clades A and B have been previously recovered from the English Channel [27], the Equatorial Pacific Ocean [2], and the Mediterranean Sea [19]. Clade VII also includes cultured strains such as CCMP1205 or RCC287 [29], although no species has yet been described formally. In the Pacific Ocean, we obtained sequences from both sub-clades A and B, and one OTU fell at the base of sub-clade B. The remaining Prasinophyceae sequences belonged to the well known Mamiellales genera *Micromonas*, *Ostreococcus*, and *Bathycoccus* (Table S2).

Among Stramenopiles, the most interesting group comprised 9 Chrysophyceae OTUs (13 full length sequences), all originating from the oligotrophic gyre and falling into three lineages (called here marine clades A, B, and C) supported by high bootstrap values, none of which contained cultured representatives. Marine clade A contained, besides sequences from the Pacific, environmental sequences from marine (Sargasso Sea and coastal Norwegian Sea) and freshwater (oligotrophic lake) ecosystems. Clone CD8.06 grouping with this lineage was found in an unamended seawater incubation in the dark and was considered to originate from a heterotrophic flagellate [30]. However, a sequence retrieved from a photosynthetic culture isolated by one of us from the Atlantic Ocean also fell into clade A (LJ, unpublished data). This lineage could therefore contain both auto- and hetero-trophic organisms, or indeed members of this lineage could be mixotrophs, since recent evidence points to the importance of this mode of nutrition for PPE [21]. Marine clade B was composed entirely of BIOSOPE sequences, whilst marine clade C contained one BIOSOPE sequence and environmental sequences from the coastal Pacific Ocean and from a lake (Fig. 3). Other photosynthetic Stramenopiles sequences belonged to diatoms, Dictyochophyceae and Pelagophyceae (Table S2). While for the first two classes, similarity to known sequences was weak, all Pelagophyceae sequences from 4 different samples formed a single OTU nearly identical to *Pelagomonas calceolata*, a species repeatedly isolated during the BIOSOPE cruise [31].

We obtained 11 OTUs of Haptophyta, most of them corresponding to the class Prymnesiophyceae, falling within 7 of the 9 clusters described by Takano et al. [32]. Some sequences were closely related to widespread genera such as *Phaeocystis* or *Emiliania* while others grouped with clades with no cultured representatives (Table S2). Interestingly, 2 OTUs originating from 2 different samples in the hyper-oligotrophic gyre, one obtained from surface waters and one from the DCM, formed a novel Haptophyta lineage with 100% bootstrap support, different from the two previously described classes of Pavlophyceae and Prymnesiophyceae (Fig. 3). This lineage could constitute a novel class within Haptophyta.

### PPE Assemblages in the South East Pacific

The composition of the PPE community was highly variable both horizontally and vertically throughout the South East Pacific (Table 2). While PPE populations display quite uniform properties when analyzed by flow cytometry and therefore are usually amalgamated as a single functional group [20], our data demonstrate that in most samples, the PPE population is in fact an assemblage of several phylogenetic groups. Two notable exceptions are constituted by samples from the Chile upwelling and from surface waters of station STB7 where a single algal order (Mamiellales) or clade (Prasinophyceae clade IX) dominated, respectively, the PPE population (Fig. 2, Table 2). Still, in the upwelling, at least two or three Mamiellales genera co-occurred in a single sample (Table S2) while in the surface layer at station STB7 at least 4 different phylotypes were observed within Prasinophyceae clade IX (Fig. 4). Such diversity within each PPE population as well as the observed spatial variability points to quite complex ecological optima for each phylotype.

Our data point to the importance of Prasinophyceae among oceanic PPE. Until recently this had only been established in coastal waters where Mamiellales [33] and especially *Micromonas* clades A and B [34] are always very important. *Micromonas* clade C, *Bathycoccus*, and *Ostreococcus* are also consistently found in coastal waters and more sporadically in pelagic waters [34], [35]. Indeed, these three genera were observed in the coastal upwelling off Chile (stations UPW1 and UPX1), where their characteristic pigments (prasinoxanthin and chlorophyll *b*) were observed [36]. The dominance of Mamiellales in flow sorted PPE samples from the upwelling where they are expected to be important indeed validates our approach. The edges of the South East Pacific gyre (e.g. STB1 and STB17) were characterized near the surface by a mixed PPE community dominated by Prasinophyceae clades VII with minor contributions from other groups (Fig. 2, Table 2). In surface waters, the contribution of clade VII decreased towards more oligotrophic waters (e.g. STA14 and STB12, Fig. 2, Table 2). Clade VII was also important at the DCM near the edge of the gyre (STB1 and STA14). This suggests that Prasinophyceae clade VII is characteristic of mesotrophic and mildly oligotrophic waters, which fits well with the fact that its sequences have been recovered from waters with similar trophic status in the equatorial Pacific Ocean [2] and in the western Mediterranean Sea [19]. This may also explain the relative ease of isolating cultures from this clade, including during the BIOSOPE cruise [31]. Prasinophyceae clade IX clearly replaced clade VII in surface waters in the central gyre (Fig. 2, Table 2) suggesting that the former prefers oligotrophic to extremely oligotrophic waters. This fits with previous observations of this clade in the very oligotrophic waters of the Eastern Mediterranean Sea [19] and may explain why it has not been brought into culture yet, since oligotrophic species are often fastidious growers [37]. The importance of Prasinophyceae is also reinforced by the fact that several plastid 16S rRNA gene sequences obtained during the BIOSOPE cruise from filtered picoplankton samples belonged to Prasinophyceae, some to clade VII and some to a novel clade (16S VIII) that could correspond to clade IX for the 18S rRNA gene [25].

In the central gyre Chrysophyceae were clearly one key component of the PPE community in surface waters (Fig. 2, Table 2). They were also present at the DCM in the western part of the gyre (STB1 and STB7) but less prevalent. This corroborates recent data based on the plastid 16S rRNA gene, both from sequencing and dot blot hybridization with specific probes which suggested that photosynthetic Chrysophyceae could be important in some marine ecosystems [38]. Indeed application of the same Chrysophyceae 16S rRNA gene probe to filtered picoplankton samples from the BIOSOPE cruise yielded strong signals in the central Pacific gyre [25]. Our 18S rRNA gene data suggests that marine Chrysophyceae are probably highly diversified. Very few marine photosynthetic Chrysophyceae have been described so far, this class being rather characteristic of freshwater ecosystems. The only major marine group assigned to this class, the Parmales, is solely known from scanning electron microscopy of natural samples [39] and no sequences are available to date. Parmales are characterized by silicified scales and only found sporadically in the ocean, most often in sub-polar waters where they can be abundant [40], but also in Pacific tropical waters [41]. Whether some of the sequences we obtained correspond to Parmales will have to wait until their 18S rRNA gene sequences become available.

The presence of Haptophyta in many samples and in particular at the DCM in the gyre (Fig. 2, Table 2) is consistent with the importance of 19'hexanoyloxyfucoxanthin in open ocean waters [42], especially in the small size classes where it can represent from 50 to more than 80% of the carotenoids [16]. Indeed in the South East Pacific gyre, 19'hexanoyloxyfucoxanthin is the major eukaryotic carotenoid [36] and many plastid 16S rRNA sequences related to Prymnesiophyceae have been recovered from <3 µm filtered samples [25]. Surprisingly, Haptophyta sequences occur in general in very low proportion in 18S rRNA gene clone libraries constructed from filtered picoplankton [16]. It has been recently argued that this low proportion was linked to the higher GC% of the rRNA gene [42] resulting in poor amplification when using universal primers. However this explanation does not seem to hold since the GC% of the 18S rRNA gene in our sorted populations is only marginally higher for Haptophyta compared to the other groups (Table 3). Also, universal primers of the 18S rRNA gene (Euk328 and Euk329) match perfectly the genomic sequence of the haptophyte *Emiliania huxleyi* that has been recently made publicly available (http://genome.jgi-psf.org/Emihu1/Emihu1.download.ftp.html). Therefore, primer mismatch cannot explain poor amplification. It is clear however, that 18S rRNA genes from haptophytes are more easily amplified with general primers when they face fewer competing templates as in the sorted samples. The nature of the picoplanktonic Haptophyta remains mysterious since very few described species from this group have a size below 3 µm [9].

**Table 3:** GC% (Mean and SD) of the partial 18S rRNA gene sequences for the different phylogenetic groups recovered (only groups for which 5 or more sequences have been obtained are considered).

Division	Class	Order	N	GC% Mean	GC% SD
Haptophyta	Prymnesiophyceae		24	0.498	0.010
Chlorophyta	Prasinophyceae	clade VIIB	29	0.491	0.008
Stramenopiles	Pelagophyceae		7	0.480	0.004
Chlorophyta	Prasinophyceae	Mamiellales	45	0.476	0.018
Chlorophyta	Prasinophyceae	Clade VIIA	13	0.475	0.007
Chlorophyta	Prasinophyceae	Clade IXB	51	0.473	0.008
Alveolata	Dinophyceae		6	0.466	0.003
Radiolaria			6	0.460	0.009
Alveolata	Dinophyceae	Syndiniales	110	0.451	0.016
Stramenopiles	Chrysophyceae	Clade marine C	5	0.439	0.027
Stramenopiles	Chrysophyceae	Clade marine A	11	0.432	0.015
Stramenopiles	Chrysophyceae	Clade marine B	17	0.416	0.013

The distribution of the other groups is too sporadic to draw major conclusions. However the case of Pelagophyceae is interesting. All sequences belonged to the same OTU and were observed over a range of stations, mostly near the DCM (Fig. 2, Table 2). The corresponding species, *P. calceolata*, has been isolated repeatedly during the BIOSOPE cruise in particular from deep stations (e.g. 4 strains were obtained from STB14 at 150 m [31]). This species constitutes with Mamiellales (e.g. *Micromonas*) a rare case where culturing and molecular data match each other.

### Comparison of Approaches to Study PPE Diversity

The two approaches used to analyze the diversity and distribution of PPE in the South East Pacific, flow cytometry sorting based on size and chlorophyll content (this work) and analysis of the plastid 16S rRNA gene on <3 µm filtered samples [25], yield remarkably similar images. Qualitatively both approaches uncover the importance of novel clades of Prasinophyceae, Chrysophyceae and Haptophyta. Quantatively, signals from probes targeting plastid 16S rRNA genes and relative abundance of 18S rRNA clones match pretty well. For example, at station STB11 both approaches suggest Chrysophyceae to be dominant in surface and Haptophyta near the DCM. The advantage of the plastid approach is that it can be performed on filtered samples that are easy to obtain on oceanographic cruises, while its main drawback is the lack of a large reference sequence database making sequence assignment sometimes difficult. Also primers and probes would need to be improved since some groups such as the Mamiellales, important in coastal waters, are apparently not well amplified or probed on natural populations [18]. The sorting approach requires the use of sophisticated and expensive flow cytometers that are challenging to use on-board ships. It has the advantage of providing full length 18S sequences which benefit from a very large reference database and allow better phylogenetic reconstruction. Also other genes can be amplified in parallel on the sorted populations (e.g. plastid 16S rRNA, X.L.S. unpublished data) and even whole genomes using Multiple Displacement Amplification [43].

### Conclusion

Flow cytometric sorting proved to be a key advance to analyze the PPE community which makes more than 40% of the phytoplankton carbon biomass in the South East Pacific [26]. This approach produced a notable reduction in the contribution of heterotrophic groups within 18S rRNA gene clone libraries and allowed the recovery of several novel lineages. The PPE community from the South East Pacific proved to be extremely diverse and variable along both horizontal and vertical gradients. Our next challenges would be (1) to establish cultures from uncultivated groups such as Prasinophyceae clade IX and (2) to obtain functional information that could explain their observed distribution.

## Materials and Methods

### Sampling

Sampling was performed in the surface layer and at the vicinity of the DCM at selected stations between 26 October and 11 December 2004 along a transect through the South East Pacific Ocean (Fig. 2, Table 1) during the BIOSOPE cruise on board the French research vessel L'Atalante. Seawater samples were collected using Niskin bottles mounted on a CTD frame. Samples were concentrated between 5 and 100-fold by tangential flow filtration using a 100 000 MWCO (Regenerated Cellulose- RC ref VF20C4) Vivaflow 200 cassette. In a methodological study done in English Channel waters [22], recovery of pico-eukaryotes after tangential flow filtration was demonstrated to range from 40 to 72%.

### Flow Cytometry Analysis and Sorting

Concentrated samples were analyzed on board using a FACSAria flow cytometer (Becton Dickinson, San Jose, CA, USA) equipped with a laser emitting at 488 nm and the normal filter setup. The signal was triggered on the red fluorescence from chlorophyll. PPE were discriminated based on side scatter, as well as orange and red fluorescence (Fig. 1), and sorted in :“purity” mode. Cells were collected into two Eppendorf tubes and, after a quick centrifugation, the volume of sorted samples was adjusted to 250 µL by adding filtered seawater. Samples were deep frozen in liquid nitrogen.

### DNA Extraction, PCR Reaction and Cloning

DNA from the sorted pico-eukaryote population was extracted using DNeasy blood and tissue kit (Qiagen), as recommended by the manufacturer. The 18S rRNA gene was amplified by the polymerase chain reaction (PCR) using the primer set Euk328f and Euk329r [27]. The PCR mixture (30 µL final volume) contained 5 µL of extracted DNA with 0.5 µM final concentration of each primer and 15 µL HotStar Taq® *Plus* Master Mix (Qiagen). PCR reactions were performed as described previously [19] with an initial incubation step at 95°C during 5 min for the activation of the HotStar Taq *Plus* DNA Polymerase. For samples for which the PCR yield was too low to allow cloning (Table 1), a second nested PCR was performed using primers Euk1A [44] and 1492rE [45] using 1 µL of a 1∶10 dilution of the first PCR product as template. Thirty-five amplification cycles were carried out as follows: 94°C for 45 s, 45°C for 45 s, and 72°C for 1 min 15 s, with the same temperature and time as the first PCR for polymerase activation and extension. Purified PCR products were cloned into vector pCR®2.1-TOPO® and transformed into *E. coli* competent cells following the manufacturer's instructions (Invitrogen, Carlsbad, California).

### Sequencing

Clone inserts were amplified with the same primers as above and purified. Partial sequences were determined from purified PCR products by using Big Dye Terminator V3.1 (Applied Biosystems, Foster city, CA, USA) and the internal primer Euk528f [27] run on an ABI prism 3100 sequencer (Applied Biosystems). Partial sequences were clustered into distinct OTUs with Clusterer [46] using a similarity threshold of 98% corresponding to the average similarity within species [28]. We obtained full length sequences for representative clones belonging to OTUs that appeared new or interesting (e.g. Prasinophyceae clade IX or Chrysophyceae) using primers M13R and M13F from the cloning kit as well as Euk528f. Sequences have been deposited to the GenBank database under accession numbers FJ537298–FJ537704.

### Sequence Analysis

Partial and full length sequences were compared to those available in public databases with the NCBI BLAST web application (May 2008, Tables S2 and S3). Sequences were analyzed with KeyDNAtools (http://keydnatools.com/), an application which provides taxonomic affiliation and chimera detection (Table S1) based on sequence motifs [5]. Sequences were aligned with related sequences from public databases using the slow and iterative refinement method FFT-NS-I with MAFFT [47] 5.8 software (http://align.bmr.kyushu-u.ac.jp/mafft/online/server/). Poorly aligned and very variable regions of the alignments were automatically removed with Gblocks [48] using the following parameters: allowing gap positions equal to “with half”, minimum length of block equal to 5 for the general analysis. Different nested models of DNA substitution and associated parameters were estimated using Modeltest [49]. Each alignment was analyzed by Maximum Parsimony (MP), Neighbour Joining (NJ) and Maximum Likelihood (ML) using PAUP 4.0b10 [50]. A heuristic search procedure using the tree bisection/reconnection branch swapping algorithm was performed to find the optimal ML tree topology (with 70,000 rearrangements). Bootstrap values for NJ and MP were estimated from 1000 replicates.

## Supporting Information

Table S1 List of potential chimeras (not considered in the final analysis).
(0.01 MB XLS)

Table S2 Partial sequences obtained from BIOSOPE sorted samples (Fungi, Metazoa and chimeras excluded). OTU assignment is based on 98% similarity: the first column indicates whether the sequence represents an OTU; the second and third columns indicate the clone library and clone number of the representative sequence of the OTU to which the sequence belongs. Taxonomic assignments have been made on the combined information from BLAST and KeyDNATools (see Methods). A sequence has been assigned to a genus if its similarity to a cultured strain belonging to this genus is higher than 98%.
(0.11 MB XLS)

Table S3 Full sequences obtained from BIOSOPE sorted samples. The OTU column indicates whether the sequence represents an OTU (see Table S2). Taxonomic assignments have been made on the combined information from BLAST and KeyDNATools (see Methods). A sequence has been assigned to a genus if its similarity to a cultured strain belonging to this genus is higher than 98%.
(0.02 MB XLS)
